# MEMO—A Mobile Phone Depression Prevention Intervention for Adolescents: Development Process and Postprogram Findings on Acceptability From a Randomized Controlled Trial

**DOI:** 10.2196/jmir.1857

**Published:** 2012-01-24

**Authors:** Robyn Whittaker, Sally Merry, Karolina Stasiak, Heather McDowell, Iain Doherty, Matthew Shepherd, Enid Dorey, Varsha Parag, Shanthi Ameratunga, Anthony Rodgers

**Affiliations:** ^1^Clinical Trials Research UnitUniversity of AucklandAucklandNew Zealand; ^2^Department of Psychological MedicineUniversity of AucklandAucklandNew Zealand; ^3^Auckland District Health BoardAucklandNew Zealand; ^4^Learning Technology UnitUniversity of AucklandAucklandNew Zealand; ^5^Injury Prevention Research UnitUniversity of AucklandAucklandNew Zealand; ^6^George Institute for Global HealthUniversity of SydneySydneyAustralia

**Keywords:** Mobile phone, depression prevention, adolescents, cognitive behavioral therapy

## Abstract

**Background:**

Prevention of the onset of depression in adolescence may prevent social dysfunction, teenage pregnancy, substance abuse, suicide, and mental health conditions in adulthood. New technologies allow delivery of prevention programs scalable to large and disparate populations.

**Objective:**

To develop and test the novel mobile phone delivery of a depression prevention intervention for adolescents. We describe the development of the intervention and the results of participants’ self-reported satisfaction with the intervention.

**Methods:**

The intervention was developed from 15 key messages derived from cognitive behavioral therapy (CBT). The program was fully automated and delivered in 2 mobile phone messages/day for 9 weeks, with a mixture of text, video, and cartoon messages and a mobile website. Delivery modalities were guided by social cognitive theory and marketing principles. The intervention was compared with an attention control program of the same number and types of messages on different topics. A double-blind randomized controlled trial was undertaken in high schools in Auckland, New Zealand, from June 2009 to April 2011.

**Results:**

A total of 1348 students (13–17 years of age) volunteered to participate at group sessions in schools, and 855 were eventually randomly assigned to groups. Of these, 835 (97.7%) self-completed follow-up questionnaires at postprogram interviews on satisfaction, perceived usefulness, and adherence to the intervention. Over three-quarters of participants viewed at least half of the messages and 90.7% (379/418) in the intervention group reported they would refer the program to a friend. Intervention group participants said the intervention helped them to be more positive (279/418, 66.7%) and to get rid of negative thoughts (210/418, 50.2%)—significantly higher than proportions in the control group.

**Conclusions:**

Key messages from CBT can be delivered by mobile phone, and young people report that these are helpful. Change in clinician-rated depression symptom scores from baseline to 12 months, yet to be completed, will provide evidence on the effectiveness of the intervention. If proven effective, this form of delivery may be useful in many countries lacking widespread mental health services but with extensive mobile phone coverage.

**ClinicalTrial:**

Australia New Zealand Clinical Trials Registry (ACTRN): 12609000405213; http://www.anzctr.org.au/trial_view.aspx?ID=83667 (Archived by WebCite at http://www.webcitation.org/64aueRqOb)

## Introduction

In 1990, depressive disorder was ranked fourth in the estimate of global disease burden. It has been predicted that it will be the second most important cause of lost healthy life-years globally by 2020 [1]. Depressive disorder commonly starts in adolescence, and its effect on young people is pervasive with respect to overall development [2-5]. It is associated with poor academic functioning, social dysfunction, substance use, and attempted and completed suicide [2,6,7]. Comorbidity is high, with up to half of those with major depressive disorder having a lifetime occurrence of another psychiatric disorder. In New Zealand, depressive disorder is a major health issue among adolescents with rates of 4%–8% at the age of 15 years rising rapidly to 17%–18% by the age of 18 years [8]. Rates are higher for young people of Maori (the indigenous population of New Zealand), Pacific, and some Asian ethnic groups [9-11].

The marked increase in period prevalence estimates from mid to late adolescence makes this a good time to intervene to prevent the onset of depressive disorder. Psychological interventions such as cognitive behavioral therapy (CBT) have been shown to be an effective treatment for depression in adolescents [12,13] and may be effective in preventing the onset of depressive disorder in children and adolescents [14]. In this review of predominantly CBT-based interventions, the change in depression scores was modest; however, the number needed to treat (NNT) to prevent one adolescent developing a depressive disorder was approximately 10. In comparison, over 800 people with uncomplicated hypertension must be treated for a year to prevent one stroke, and 67 people who have had a myocardial infarction need to take aspirin to prevent one death from any cause [15,16]. Reduction in depression has been shown for both interventions targeted at those at risk (NNT of 8) and universal interventions for all adolescents (NNT of 13). The most effective study reported to date was a program targeted at children whose parents had mood disorders, where the NNT was 4 [17].

A randomized placebo-controlled trial of a school-based depression prevention program (RAP-Kiwi) used manuals with instructions on CBT in graphics and text, and comprised 11 sessions delivered during the school day as one of the regular health classes for all students [18]. A total of 392 participants were recruited from two different years in two schools in Auckland, New Zealand—one from a lower socioeconomic urban area and the other from a middle-class rural district. Immediately after the program, depression scores (measured by the Beck Depression Inventory and Reynolds Adolescent Depression Scale, Second Edition [RADS-2]) were reduced significantly more by RAP-Kiwi than by placebo. Categorical analysis confirmed significant clinical benefit with an absolute risk reduction of 3% (95% confidence interval 1%–11%, McNemar χ^2^, *P* = .03) and an NNT for short-term benefit of 33. However, the considerable teaching time required and the difficulty ensuring fidelity of the program were both considered problematic with respect to a widespread rollout. These factors suggested that a new delivery medium would be necessary.

The ubiquitous spread of mobile phones allowed the consideration of an automated intervention for widespread cost-effective dissemination. Mobile phones have been successfully used to deliver behavior change interventions for smoking cessation [19], medication reminders [20-22], diet and physical activity [23-26], and the management of diabetes [27-30]. Our research team has used behavior change theory and techniques to guide the development of mobile phone interventions using text and video messages [31-33]. This research brought together this mobile health expertise with expertise from the RAP-Kiwi depression prevention research. We set out to adapt traditionally manualized and therapist-delivered CBT techniques into automated messages that could be delivered on a multimedia-capable mobile phone, with the aim of preventing the onset of depressive disorder in all New Zealand adolescents.

### Objective

The objective of the randomized controlled trial (RCT) was to test whether the mobile phone CBT intervention can improve subjective and objective scores of depression symptoms in adolescents in comparison with a control group at 12 months (to be published separately). The aim of this paper is to describe the developmental process, and the acceptability and utility of the intervention as reported by the adolescents at the end of the intervention (9 weeks).

## Methods

### Intervention Development

Initial focus groups with students (n = 27) in a low socioeconomic multicultural high school formed an understanding of how adolescents use their mobile phones and how a well-being and problem-solving program could be useful and appealing to them. All participants used mobile phones, predominantly for text messaging. Concerns were around the cost of other functions beyond text messaging and potential loss of confidentiality with video calling. In general, students felt that text messages would be useful for information and positive reinforcement, videos could be used to demonstrate strategies for dealing with problems, and music or music videos could be used for relaxation. Some initial short videos were pretested with students (n = 40) at a small predominantly indigenous high school via computer. The students’ preferences for different styles of videos and animations directly informed the development of the intervention. Once some initial content had been developed, pretesting took place during the development process with groups of adolescents (n = 28) in various environments (youth centers, libraries, and invited group sessions). A variety of different text and video messages were shown to participants on a laptop computer. Their feedback supported the use of a variety of message types, as different young people found different mediums of delivery appealing. Important themes arising from these discussions were the importance of realism, credibility, positivity, and simple, clear messages.

An expert content group with expertise in adolescent psychiatry and psychological therapies, CBT, learning technology, marketing and media, and mobile phone health interventions was formed. The group derived 15 key messages, considered appropriate for delivery by mobile phone, from CBT and from RAP-Kiwi (Textbox 1).

To enhance relevance and thereby engagement with the program, it was important to deliver these key CBT themes within everyday contexts and addressing common issues faced by New Zealand adolescents. This was also a good fit with the theoretical basis, derived from social cognitive theory and tested in our previous smoking cessation video messaging intervention [33], of enhancing self-efficacy to deal with issues and life events using cognitive techniques [34]. This led to the use of observational learning to observe other “ordinary” teens facing typical issues or events and using the techniques to feel more positive about these issues. The idea was that the observers are more likely to try the techniques if they see other people like themselves successfully using them [35-40]. For example, story lines delivered in a series of short videos illustrate how moods follow thoughts, with negative thoughts leading to low mood, and how the recognition of cognitive distortions (often by a friend) can lead to a more positive mood. Actual techniques, such as a problem-solving mnemonic (STEPS), could be normalized in the demonstration of its use by adolescents for common adolescent problems.

The mode of delivery was carefully considered: whereas some Web- and computer-based interventions have been primarily manuals adapted for use on the computer, this approach has been associated with poor adherence and was unlikely to work for mobile phones [41]. A variety of types of mobile phone messages were considered—video clips, text messages, and animated cartoons—in order to widen the appeal and potential for engagement with the target audience. In this way, the same message could be repeated in different formats to increase recall.

As well as the ordinary teen role models described above, our initial focus groups had confirmed the importance of celebrities for teenagers, and so we included video messages from celebrities. In these short video clips they discussed the same strategies for dealing with difficult times in their own lives. Animations were also popular with our formative research participants. A cartoon was developed with *mobisodes* (short episodes by mobile phone) about four fictitious adolescents—repeating the key CBT messages in a different format within a different story that might appeal to a different cohort of teenagers. Homework, trying out the techniques in their own lives, is an important part of CBT and was addressed by celebrity video messages setting relevant weekly challenges for participants.

Based on the feedback from adolescents in the focus groups and pretesting, a regimen of 2 messages per day (outside school hours) over 9 weeks was developed. Media and marketing principles were applied to the importance of overall framing of the program and to the careful design of individual text and video messages. The program was given identity and coherence with a logo and byline (MEMO: living in a positive space), theme music, and three key words (spot, sort, do). The key CBT messages were reinforced by short memorable text messages that all related to one of the three key words.

To provide ongoing contact and reminders of the themes after the 9-week intervention, monthly text messages directed participants to a mobile website. This website (available only to recognized participant mobile phone numbers) provided a summary of the key messages, information on how to get more help, and a downloadable relaxation audio. New videos were posted on the website monthly. Ringtones, wallpaper images, and music downloads were linked to the mobile website.

**The 15 key cognitive behavioral therapy-based messages that formed the basis of the MEMO intervention**

Your feelings are a result of what you think and what you do

You can take control of this

Being busy increases happiness generally

Do fun stuff

Don’t procrastinate

Relaxation makes you feel good

Being with people you like makes you feel good

Fighting with people makes you feel bad

Sometimes you need to ask for help

Noticing only weaknesses and failures makes you feel bad

It’s not what happens; it’s what you think about it that affects feelings

We can choose to look at the world in a positive or a negative way

We can deal with negative thoughts

There are ways of dealing with stress

There are problem-solving techniques

### Study Design

This research was part of a double-blind RCT (June 2009–April 2011) designed to test the effectiveness of the mobile phone intervention. Human subjects ethical approval was provided by the Northern Region Y Ministry of Health Human Ethics Committee (NTY/0/09/088).

Support from a mobile telecommunications company (Vodafone New Zealand Ltd, Auckland, New Zealand) ensured that the program was delivered completely free of cost to participants.

### Recruitment

A total of 15 high schools across Auckland city (population approximately 1.4 million) agreed to participate in the study. The included schools represented a range of private/public, single-sex/coeducation, ethnicity, and decile ratings (a school’s decile reflects the socioeconomic status of the school’s community based on the New Zealand census). Study processes were agreed on with each school guidance counselor or team. Parents of potential participants were sent participant information sheets and study contact details, and were given the opportunity to opt their child out of the study. One school required written parental consent. The research team promoted the study to large groups of students at assemblies or other organized school sessions. It was described as a study involving a mobile phone program about “living in a positive space.” Those who wished to participate were given full study information and were asked to complete a written consent form and baseline data collection forms immediately after the presentation. Students identified as having current depressive symptoms (score of 76 or higher on the self-completed RADS-2) or at risk of self-harm (question 14 on the RADS-2, “I feel like harming myself sometimes” or “often”) were immediately referred for management by the school guidance counselors according to school protocols [42]. These students were excluded from this study, as the research question was about the prevention of onset of depressive disorder.

All students completing baseline data collection received a run-in program consisting of daily mobile phone messages for 9 days. These messages provided instructions about the study and allowed students to determine whether they could view the video messages on their mobile phones (if not, they could not proceed to randomization). This run-in period was also designed to allow for the initial sharing of messages between friends without potentially contaminating randomized groups. During this period, potential participants were invited to an individual interview with a trained research assistant who conducted the Child Depression Rating Scale-Revised (CDRS-R) [43]. Again, any students exhibiting current depression or risk of self-harm were referred for management by the school guidance counselors and excluded from the study.

At this point, all those who met the eligibility criteria were randomly assigned to receive either the intervention or control program. Allocation concealment was maintained by computer-based randomization so that researchers were unaware of possible allocation. A stratified minimization was used to ensure balance for possible prognostic factors: sex, ethnicity (Maori/Pacific vs non-Maori/non-Pacific), and school. Active recruitment ceased once the target sample size had been reached—all students who had been offered participation at that stage were accepted into the study.

### Intervention and Control

Students in the intervention group then received 2 messages per day for 9 weeks (outside school hours), followed by monthly messages and access to a mobile website. The messages were a mixture of text messages, video messages of adolescents and celebrities, and animated cartoons (described above; see Multimedia Appendix 1 for an example video message). Because of the potential for high levels of placebo response in depression interventions, students in the control group received a full attention control program with the same number of mobile phone messages, the same types of messages, including the same adolescents and celebrities in the video messages, and the same characters in the cartoon messages. The content of the placebo messages was focused on healthy eating, sustainability of the environment, and safe practices for using the Internet and mobile phone (cybersafety). Participants were not aware of which program was the intervention and which was the control.

### Outcome Measures

Individual interviews were arranged with participants at the end of the 9-week program and took place on school grounds. During these interviews, participants self-completed forms on how much of the program they had viewed (as a measure of adherence to the program) and their perceived usefulness of the program. The interviews were conducted by research assistants blinded to allocation. The primary outcome for the trial will be the change in the clinician-assessed (blinded) depression symptom scores (CDRS-R) from baseline to 12 months [43]. Secondary outcomes will include change in the self-rated RADS-2 depression symptom scale [42]; incidence of depression; general and school functioning; and quality of life.

### Statistical Analysis

The target sample size for the trial was calculated for the primary RCT outcome of change in CDRS-R (not reported here) at 790 participants. This was based on randomization at the individual level, as cluster randomization by school would have required a larger sample that was not considered feasible by the research team. All analyses were 2-tailed and conducted according to a prespecified plan using SAS version 9.2 (SAS Institute, Cary, NC, USA). Continuous variables are presented as means and standard deviations, and categorical variables are presented as counts and percentages. Differences between groups for categorical outcomes were analyzed using chi-square tests or Fisher exact test.

## Results

**Figure 1:**
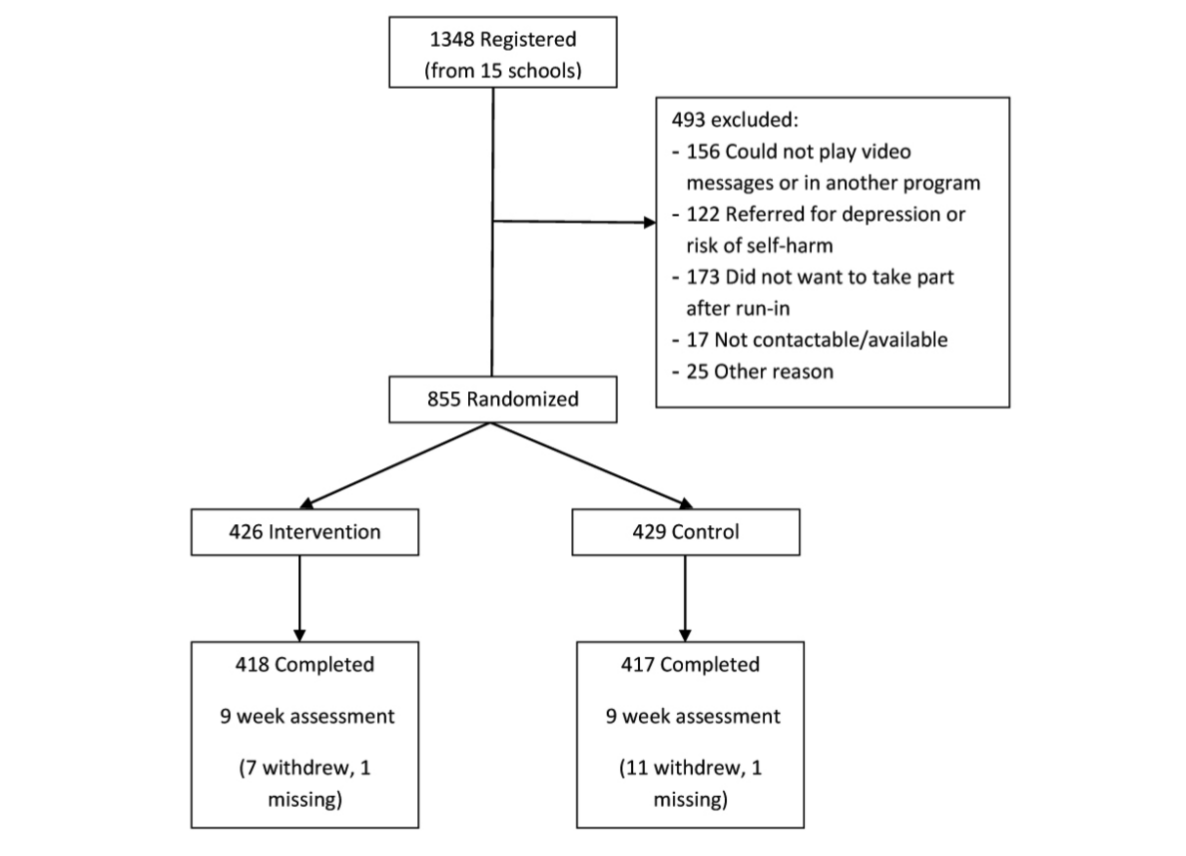
CONSORT flowchart of participants in the MEMO trial.

Figure 1 depicts the flow of participants through study processes. From an estimated 2850 students attending the presentation sessions, 1348 students (47.30%) registered initial interest in participating in the study. In three cases, parents actively opted their child out of the study. Of those registered, 122 (9.1%) were referred to the school guidance counselors for immediate management of depression or risk of self-harm. A further 371 (27.5%) dropped out at this stage due to lack of interest or difficulty viewing the video messages on their phones, or were excluded for having been recently through a school-based depression program for at-risk students. A total of 855 students (63.4% of those registered) were individually randomly assigned: 426 to the intervention group and 429 to the control group.

Of the 855 randomly assigned participants, 584 (68.3%) were female and 271 (31.7%) were male (Table 1). The largest group was New Zealand Europeans (501, 58.6%), followed by Asian students (208, 24.3%), Maori (83, 10%; the indigenous population of New Zealand who make up approximately 14% of the overall population), and Pacific students (51, 6%). This gender and ethnicity distribution mostly reflects the participating schools—for example, overall the schools in the study contained 9% Maori students and 5% Pacific students. The age range of participants is from 13 years to 17 years, with a mean age of 14 years.

Participants were asked to specify (within categories) how many of the messages they viewed. Approximately three-quarters of the intervention group (311/418, 74.4%) viewed at least half of the messages, with 29.6% (n = 123) viewing most or all of the messages (Table 2). Although more than a third (324/835, 38.8%) of participants shared messages (with anyone, not necessarily someone in the study), they mostly shared only a small number (<10) of messages. This number of messages was not considered sufficient to have an effect even if shared with someone in the other group (contamination). Participants in the intervention group seemed to like the types of messages that were used: 78.0% (326/418) liked the video messages from celebrities; 71.8% (300/418) liked the video messages from other teens; 65.8% (275/418) liked the animated cartoons; and 60.3% (252/418) liked the text messages.

**Table 1:** Baseline participant characteristics

		Intervention (n = 426)	Control (n = 429)
**Sex**, n (%)			
	Female	291 (68.3)	293 (68.3)
	Male	135 (31.7)	136 (31.7)
**Ethnicity**, n (%)			
	NZ^a^ European	245 (57.5)	256 (59.7)
	Maori (NZ indigenous population)	38 (9)	45 (11)
	Asian	113 (26.5)	95 (22)
	Pacific	23 (5)	28 (7)
	Other	7 (2)	5 (1)
Age (years), mean (SD)		14.3 (0.90)	14.3 (0.91)
**School year**, n (%)			
	Year 9 (13 & 14 years of age)	74 (17)	84 (20)
	Year 10 (14 & 15 years of age)	174 (40.9)	169 (39.4)
	Year 11 (15 & 16 years of age)	148 (34.7)	148 (34.5)
	Year 12 (16 & 17 years of age)	30 (7)	28 (7)

^a^ New Zealand.

**Table 2:** Participant viewing and sharing of messages

		Intervention (n = 418)	Control (n = 417)
**Number of m** **essages viewed**, n (%)			
	Most/all	123 (29.6)	142 (34.4)
	More than half	96 (23)	110 (26.6)
	About half	92 (22)	82 (20)
	Some	74 (18)	56 (14)
	Hardly any	30 (7)	24 (6)
	Missing data	3	3
**Number of m** **essages shared with others**, n (%)		158 (37.9)	166 (40.1)
	1–9	111 (70.3)	125 (75.8)
	10–19	37 (23)	31 (19)
	20–29	5 (3)	7 (4)
	30+	5 (3)	2 (1)

The majority of participants (688/835, 82.4%) said they found MEMO to be useful (Table 3). A significantly greater proportion of those in the intervention group (379/418, 90.7%) said they would recommend it to a friend than those in the control group (345/417, 82.7%, *P* < .001). Participants were asked whether the program helped them with particular topics—some topics from the intervention and some from the control program. More participants in the intervention group than in the control group said that MEMO helped them to be more positive, to get rid of negative thoughts, to relax, to solve problems, to have fun, and to deal with issues in school (Table 3). These make up some of the key CBT messages in the intervention. As expected, more control group participants said the program helped them with topics covered in the control program (eg, be safe on the Internet, think more about the environment). Similar proportions in both groups said the program helped them with some of the issues considered to be in the intervention only, such as ‘to help other people,” and disappointingly the intervention group participants were no more likely than the control group participants to know where to go for help.

**Table 3:** Participant satisfaction and perceived usefulness of MEMO

		Intervention (n = 418)	Control (n = 417)	χ^2^ *P* value
Found MEMO to be helpful, n (%)		351 (84.0)	337 (80.8)	.29
Would recommend MEMO to friends, n (%)		379 (90.7)	345 (82.7)	<.001
**MEMO helped me to...**, n (%)				
	be more positive	279 (66.7)	209 (50.1)	<.001
	be nicer to people	140 (33.5)	102 (24.5)	.004
	get rid of negative thoughts	210 (50.2)	135 (32.4)	<.001
	relax	222 (53.1)	176 (42.2)	.002
	solve problems	138 (33.0)	104 (24.9)	.01
	have fun	186 (44.5)	160 (38.4)	.07
	deal with issues at school	109 (26.1)	80 (19)	.02
	deal with issues at home	88 (21)	68 (16)	.08
	be healthy	112 (26.8)	233 (55.9)	<.001
	be safe on the net	68 (16)	175 (42.0)	<.001
	get support when I need it	122 (29.2)	94 (23)	.03
	be safe with my mobile phone	101 (24.2)	179 (42.9)	<.001
	know who to go to when I need help	137 (32.8)	139 (33.3)	.86
	think more about the environment	75 (18)	176 (42.2)	<.001
	help other people	177 (42.3)	160 (38.4)	.24
	speak out about things I’m passionate about	145 (34.7)	104 (24.9)	.002

More female students than male students perceived the program as being helpful (Table 4). This appears to hold true for most of the key messages except where more male students stated “MEMO helped me to relax.” Participants in the intervention group who answered that they did not find MEMO to be useful stated their reasons in free text. These were categorized as follows: it didn’t change the way I thought (n = 15); I didn’t have any problems/already happy so didn’t need any help (n = 15); I had technical difficulties (n = 15, including not viewing the videos, taking too long to download, and lost or broken phone); issues covered were too minor or not relevant (n = 10); it was boring/nothing new (n = 7); other (n = 7). The intervention group’s suggestions for improvements were overwhelmingly related to reducing the number of messages. Other suggestions included providing solutions to other (more serious) problems.

The intervention ran as intended throughout the study, but technical issues arose from the large number of overseas mobile phones (not distributed by New Zealand telecommunications companies) that did not have the appropriate New Zealand Internet settings. Research staff had to assist these students to change the settings before they could commence the program. Also the telecommunications company changed the way they charged customers for Internet access during the study period, which caused a few participants to be charged for viewing the video messages. This was addressed once the issue was identified, and the participants were reimbursed for any charges.

**Table 4:** Perceived usefulness by sex

		Intervention		Control	
		Female (n=285)	Male (n=133)	Female (n=287)	Male (n=130)
Would recommend MEMO to friends, n (%)		259 (91.0)	120 (90.2)	240 (83.6)	105 (80.8)
**MEMO helped me to** **...**, n (%)					
	be more positive	205 (71.9)	74 (56)	149 (51.9)	59 (45)
	get rid of negative thoughts	154 (54.0)	56 (42)	98 (34)	37 (29)
	relax	142 (49.8)	80 (60)	124 (43.2)	52 (40)
	solve problems	100 (35.1)	38 (29)	81 (28)	23 (18)
	know who to go to when I need help	91 (32)	46 (35)	105 (36.6)	34 (26)
	help other people	130 (45.6)	47 (35)	114 (39.7)	46 (35)

## Discussion

This study shows that key messages from CBT can be delivered by mobile phone and that young people report that these are helpful. This is the first study to deliver a CBT-based intervention to prevent depression in adolescents via mobile phone text and video messages. Feedback from New Zealand adolescents has shown that the program is acceptable, with three-quarters watching more than half of the messages and a very high proportion recommending it to their friends. Even more encouraging are the findings that participants felt MEMO helped them to be more positive, get rid of negative thoughts, relax, solve problems, and deal with issues at school. More work could be done to refine the intervention for the future; in particular, participants suggested that the number of messages be reduced. Further qualitative research with adolescents is also underway to obtain more in-depth feedback on the intervention, and to determine its appeal to different subgroups and how to improve it.

Limitations of this study include the use of self-reported outcomes from adolescents. As participants did not know whether the program they received was the intervention or the control program, this is unlikely to have a major effect on the findings. We did not measure possible mediators such as self-efficacy (from the theoretical base), nor did we ask participants whether they had tried any of the techniques suggested. Qualitative research conducted after the RCT may address some of these and will be reported separately. It is not clear how generalizable these results will be for other populations. In particular, it is disappointing for us that the trial did not recruit a large proportion of young Maori participants. However, this was representative of the schools involved in the study, and in terms of absolute numbers this compares favorably with other studies of CBT-related interventions with indigenous populations. Two other major ethnic minority groups appear to be reasonably well represented (Pacific people comprise 6.9%, and Asian people 9.2%, of the New Zealand population, according to Statistics New Zealand).

Primary outcomes from the RCT will be required to determine the overall effectiveness of the intervention with respect to the prevention of depression symptoms at 12 months postrandomization. If the encouraging results presented here are maintained, mobile phone programs could be a cost-effective method for delivering basic CBT techniques to all adolescents. Such programs can be easily scaled up to reach large disparate populations regardless of geographic location, as has been shown by the implementation of a successful text message smoking cessation intervention [31] as a free national program in New Zealand [44]. Promotion through schools is one option, as shown in the study, but other distribution options may also be possible. Also, the development concepts and key messages in this intervention may translate to other populations with the adaptation of local content. With the increasing prevalence of mobile phone use in low- and middle-income countries and the use of mobile health technologies to address many aspects of noncommunicable diseases, using strategies of this kind has significant potential to address global disparities in the burden of adolescent depression.

## Multimedia Appendix 1

MEMO example video message of a New Zealand teen talking about their video diary.

## Multimedia Appendix 2

CONSORT-EHEALTH (V1.6) checklist [45].

## Abbreviations

CBT: cognitive behavioral therapy
CDRS-R: Child Depression Rating Scale-Revised
NNT: number needed to treat
RADS-2: Reynolds Adolescent Depression Scale, Second Edition
RCT: randomized controlled trial
