# Supplementary material for: MEMO—A Mobile Phone Depression Prevention Intervention for Adolescents: Development Process and Postprogram Findings on Acceptability From a Randomized Controlled Trial
Source: J Med Internet Res. 2012 Jan 24;14(1):e13. doi: 10.2196/jmir.1857 (PMC3846345; doi:10.2196/jmir.1857)
Supplement: Supplementary file 2 [file jmir_v14i1e13_app2.pdf]

CONSORT-EHEALTH Checklist V1.6 Report

Manuscript Number 1857

Date completed 8/25/2011 14:50:29

by Robyn Whittaker

MEMO
- a mobile phone depression prevention intervention for adolescents: development process and post-program findings on acceptability from a randomized controlled trial.

TITLE

1a-i) Identify the mode of delivery in the title

"MEMO
- a mobile phone depression prevention intervention for adolescents: development and post-program findings from a randomized controlled trial."

1a-ii) Non-web-based components or important co-interventions in title

this intervention was delivered solely over the mobile phone so no further information is added

1a-iii) Primary condition or target group in the title

MEMO
- a mobile phone "depression prevention" intervention for "adolescents": development and post-program findings from a randomized controlled trial.

ABSTRACT

1b-i) Key features/functionalities/components of the intervention and comparator in the METHODS section of the ABSTRACT

"The intervention was developed based on 15 key messages derived from cognitive behavioral therapy (CBT). The program was fully automated and was delivered in 2 mobile phone messages/day for 9 weeks, with a mixture of text, video and cartoon messages and a mobile website. Delivery modalities were guided by social cognitive theory and marketing principles. This was compared with an attention control program of the same number and types of messages on different topics."

1b-ii) Level of human involvement in the METHODS section of the ABSTRACT

"The program was fully automated and..."

1b-iii) Open vs. closed, web-based (self-assessment) vs. face-to-face assessments in the METHODS section of the ABSTRACT

"students (13-17 years of age) volunteered to participate at group sessions in schools, and 855 were eventually randomized. Of these, 835 (97.7%) self-completed follow-up questionnaires at post-program interviews on satisfaction, perceived usefulness, and adherence to the intervention."

1b-iv) RESULTS section in abstract must contain use data

"A total of 1348 students (13-17 years of age) volunteered to participate at group sessions in schools, and 855 were eventually randomized. Of these, 835 (97.7%) self-completed follow-up questionnaires at post-program interviews on satisfaction, perceived usefulness, and adherence to the intervention. Over three-quarters of participants viewed at least half of the messages and 91% reported they would refer the program to a friend. Intervention group participants said the intervention helped them to be more positive (66.7%) and get rid of negative thoughts (50.2%)"

1b-v) CONCLUSIONS/DISCUSSION in abstract for negative trials

INTRODUCTION

2a-i) Problem and the type of system/solution

"depressive disorder was ranked fourth in the estimate of global disease burden. It has been predicted to be the second most important cause of lost healthy life years globally by 2020 [1]. Depressive disorder has a high rate of relapse and commonly starts in adolescence [2-5]. In young people the effect of depressive disorder is pervasive and affects not only function but overall development. Depressive disorder is associated with poor academic functioning, social dysfunction, substance use, attempted and completed suicide [2,6,7]. Co-morbidity is high, with up to half of those with major depressive disorder having a life time occurrence of another psychiatric disorder."
"the program was quite resource intensive since it relied on teachers to deliver the program. Ensuring adherence of the teachers to the program with widespread dissemination is also problematic. These factors suggested a new delivery medium would be of benefit.... A fully automated mobile intervention could allow widespread dissemination of a standard program in a more cost-effective manner."

2a-ii) Scientific background, rationale: What is known about the (type of) system

"A randomized placebo controlled trial of a school-based depression prevention program (RAP Kiwi) used manuals with instructions on CBT in graphics and text, and comprised 11 sessions delivered during the school day as one of the regular health classes [18].....Mobile phones have been successfully used to deliver behavior change interventions for smoking cessation [19], medication reminders [20-22], diet and physical activity [23-26], and the management of diabetes [27-30]. Our research team has used behavior change theory and techniques to guide the development of mobile phone interventions using text and video messages [31-33]."
The justification of the choice of comparator is discussed later in the manuscript

METHODS

3a) CONSORT

"The
objective of the randomized controlled trial is to test whether the mobile phone CBT intervention can improve subjective and objective scores of depression symptoms in adolescents in comparison with a control group at twelve months. The aim of this paper is to describe the developmental process, and the acceptability and utility of the intervention as reported by the adolescents at the end of the intervention (9 weeks). "

3b-i) Bug fixes, Downtimes, Content Changes

"The
intervention ran as intended throughout the study, however technical issues arose from the large number of overseas mobile phones (imported other than by the telecommunications companies) that did not have the appropriate New Zealand internet settings. Research staff had to assist these students to change the settings before they could commence the program. Also the telecommunications company changed the way they charged customers for internet access during the study period, which caused a small number of participants to be charged for viewing the video messages. This was addressed once the issue was identified, and the participants reimbursed for any charges."

4a-i) Computer / Internet literacy

internet literacy not required

4a-ii) Open vs. closed, web-based vs. face-to-face assessments:

"The
research team promoted the study to large groups of students at assemblies or other organized school sessions as involving a mobile phone program about “living in a positive space”. Those who wished to participate were given full study information and asked to complete a written consent form and baseline data collection forms immediately after the presentation." "During this period, potential participants were invited to an individual interview with a trained Research Assistant (RA) who conducted the Child Depression Rating Scale (CDRS-R) [43]. " "The outcomes analyzed here are those available from the post-program assessment interview at nine weeks. These include a self-completed questionnaire on satisfaction, perceived usefulness of the program, and the approximate number of messages viewed as a surrogate for ‘adherence’ to the program."

4a-iii) Information giving during recruitment

"Parents
of potential participants were sent participant information sheets, study contact details, and given the opportunity to opt their child out of the study. One school required written parental consent. The research team promoted the study to large groups of students at assemblies or other organized school sessions as involving a mobile phone program about “living in a positive space”. Those who wished to participate were given full study information and asked to complete a written consent form and baseline data collection forms immediately after the presentation"

4b-i) Report if outcomes were (self-)assessed through online questionnaires

"The
outcomes analyzed here are those available from the post-program assessment interview at nine weeks. These include a self-completed questionnaire on satisfaction, perceived usefulness of the program, and the approximate number of messages viewed as a surrogate for ‘adherence’ to the program."

4b-ii) Report how institutional affiliations are displayed

Not important here

5-i) Mention names, credential, affiliations of the developers, sponsors, and owners

All developers/researchers are university employees and therefore do not have any conflicts of interest or stand to benefit personally from this intervention. Their affiliations are included.

5-ii) Describe the history/development process

"Initial focus groups with students (n=27) in a multicultural low socioeconomic high school formed an understanding of how adolescents use their mobile phones and how a “wellbeing and problem solving” program could be useful and appealing to them. All students used mobile phones, predominantly for text messaging. Concerns were around the cost of other functions beyond text messaging and loss of confidentiality with video calling. In general, students felt that text messages would be useful for information and positive reinforcement, videos could be used to demonstrate strategies for dealing with problems, and music/music videos could be used for relaxation. Some initial video content was pre-tested with students (n=40) at a small predominantly indigenous high school via computer. The students’ feedback on preferences and styles directly informed the further development of the intervention.

An expert content group with expertise in adolescent psychiatry and psychological therapies, cognitive behavioral therapy, learning technology and mobile phone health interventions was formed to develop the intervention. Fifteen key messages that were considered appropriate for delivery by mobile phone were derived from CBT (Box 1). The mode of delivery of cognitive therapy constructs needed to be reconsidered: whereas some web and computer based interventions are primarily manuals adapted for use on the computer, this approach has been associated with poor adherence and was unlikely to work for mobile phones [34]. A variety of mobile phone message types could be delivered -video clips, text messages, and even animated cartoons – in order to widen the appeal and potential for engagement with the target audience. Given the range of modalities the same message could be repeated in different formats to increase recall. Marketing and media advisors on the expert content group provided input into the careful design of individual text and video messages, as well as advice on the importance of the overall framing of the program. ..."

5-iii) Revisions and updating

Revisions were only made during the intervention development phase as described above. Once the study started no further revisions were made

5-iv) Quality assurance methods

Im not quite sure what this question refers to - QA methods around the information within the intervention? QA methods for collected information?

5-v) Ensure replicability by publishing the source code, and/or providing screenshots/screen-capture video, and/or providing flowcharts of the algorithms used

Im not exactly sure how this is appropriate here as this is a huge and complex intervention, also the university has rules about the sharing of some of this information

5-vi) Digital preservation

There is no URL

5-vii) Access

The intervention was only available to participants in the study - eligibility and consent process are described in the paper.
"Support from a mobile telecommunications company (Vodafone New Zealand Ltd) ensured that the program was delivered completely free of costs to participants. "

5-viii) Mode of delivery, features/functionalities/components of the intervention and comparator, and the theoretical framework

There is a whole section in the manuscript on intervention development that describes the different components of the intervention and theoretical basis

5-ix) Describe use parameters
"A regimen was developed by the investigators for the delivery of the program in two messages per day (outside school hours) for nine weeks."
5-x) Clarify the level of human involvement
The intervention is completely automated, personal involvement was only for data collection for the study and this is explained in the manuscript
5-xi) Report any prompts/reminders used
nil
5-xii) Describe any co-interventions (incl. training/support)
nil
6a-i) Online questionnaires: describe if they were validated for online use and apply CHERRIES items to describe how the questionnaires were designed/deployed
no online questionnaires
6a-ii) Describe whether and how "use" (including intensity of use/dosage) was defined/measured/monitored
"Participants were asked to specify (within categories) how many of the messages they viewed. Approximately three-quarters of the intervention group viewed at least half of the messages, with 30% viewing most or all of the messages (Table 2). " Categories are shown in the table.
6a-iii) Describe whether, how, and when qualitative feedback from participants was obtained
"These include a self-completed questionnaire on satisfaction, perceived usefulness of the program, and the approximate number of messages viewed as a surrogate for 'adherence' to the program. "
7a-i) Describe whether and how expected attrition was taken into account when calculating the sample size
"The target sample size for the trial was set at 790 in order to detect a 3 point change in CDRS-R score at 12 months (SD=8.6) with over 90% power, assuming 20% loss to follow-up and p=0.05. "
7b) CONSORT
no interim analyses. There were stopping guidelines which will be explained in the full results paper where this is more appropriate
8a) CONSORT
"Allocation concealment was maintained by computer-based randomization so that researchers were unaware of possible allocation. A stratified minimization was used to ensure balance for possible prognostic factors: sex, ethnicity (Maori/Pacific v. non-Maori/non-Pacific) and school. "
8b) CONSORT
"Allocation concealment was maintained by computer-based randomization so that researchers were unaware of possible allocation. A stratified minimization was used to ensure balance for possible prognostic factors: sex, ethnicity (Maori/Pacific v. non-Maori/non-Pacific) and school. "
9) CONSORT
"Allocation concealment was maintained by computer-based randomization so that researchers were unaware of possible allocation. A stratified minimization was used to ensure balance for possible prognostic factors: sex, ethnicity (Maori/Pacific v. non-Maori/non-Pacific) and school. "
10) CONSORT
"Allocation concealment was maintained by computer-based randomization so that researchers were unaware of possible allocation."
"The research team promoted the study to large groups of students at assemblies or other organized school sessions as involving a mobile phone program about "living in a positive space". Those who wished to participate were given full study information and asked to complete a written consent form and baseline data collection forms immediately after the presentation. "
11a-i) Specify who was blinded, and who wasn't
"Follow-up interviews with participants were undertaken by RAs blinded to allocation."
11a-ii) Discuss e.g., whether participants knew which intervention was the "intervention of interest" and which one was the "comparator"
" participants were not aware which program was the intervention and which was the control.""
11b) CONSORT
"Students in the control group were provided with a full attention control program (of the same frequency and type of messages, to be described elsewhere) "
12a) CONSORT
"Basic descriptive analyses were used on the post-program feedback and the chi-squared test was used to compare the difference between groups where appropriate. "
12a-i) Imputation techniques to deal with attrition / missing values
this was not relevant for the satisfaction results presented here.
12b) CONSORT
Not relevant here

RESULTS

13a) CONSORT
A consort flowchart is included
13b) CONSORT
The small numbers who withdrew or were missing are included in the consort flowchart, reasons are not known
13b-i) Attrition diagram
A table on the number of messages viewed by participants is included
14a) CONSORT
June 2009 - April 2011
14a-i) Indicate if critical "secular events" fell into the study period
Nil of note
14b) CONSORT
not applicable
15) CONSORT
table included
15-i) Report demographics associated with digital divide issues
age and gender included, education and SES not appropriate
16-i) Report multiple "denominators" and provide definitions
Ns included in tables
16-ii) Primary analysis should be intent-to-treat
Yes
17a) CONSORT
Primary outcomes not reported here
17a-i) Presentation of process outcomes such as metrics of use and intensity of use
Participants report on number of messages viewed reported (as before)
17b) CONSORT
pimary outcomes not reported
18) CONSORT
Primary outcomes not reported
18-i) Subgroup analysis of comparing only users
not applicable
19) CONSORT
To be reported with primary outcomes paper
19-i) Include privacy breaches, technical problems

"The intervention ran as intended throughout the study, however technical issues arose from the large number of overseas mobile phones (imported other than by the telecommunications companies) that did not have the appropriate New Zealand internet settings. Research staff had to assist these students to change the settings before they could commence the program. Also the telecommunications company changed the way they charged customers for internet access during the study period, which caused a small number of participants to be charged for viewing the video messages. This was addressed once the issue was identified, and the participants reimbursed for any charges."

19-ii) Include qualitative feedback from participants or observations from staff/researchers

Yes these are rported

DISCUSSION

20-i) Typical limitations in ehealth trials

This will be presented with primary outcomes

"Limitations of this paper include the use of self-reported outcomes from adolescents."

21-i) Generalizability to other populations

"It is not clear how generalizable these results will be for other populations. The trial did not recruit a large proportion of young Maori participants. However, this was representative of the schools involved in the study, and in terms of absolute numbers this compares favorably with other studies of CBT-related interventions with indigenous populations. Two other major ethnic minority groups appear to be reasonably well represented (Pacific people comprise 6.9%, and Asian people 9.2%, "

21-ii) Discuss if there were elements in the RCT that would be different in a routine application setting

Data collection would not be repeated in routine application, this will be discussed in detail with the primary outcomes

22-i) Restate study questions and summarize the answers suggested by the data, starting with primary outcomes and process outcomes (use)

"This study shows that key messages from cognitive behavioral therapy can be delivered by mobile phone and that young people report that these are helpful. "

22-ii) Highlight unanswered new questions, suggest future research

"Primary outcomes from the randomized controlled trial will be required to determine the overall effectiveness of the intervention with respect to the prevention of depression symptoms at twelve months post-randomization. "

Other information

23) CONSORT

"Australia & NZ Clinical Trials Registry: ACTRN12609000405213"

24) CONSORT

we have not published the protocol but would be happy to do so

25) CONSORT

"This research was supported by the Health Research Council of New Zealand, the Oakley Mental Health Research Foundation, the University of Auckland Faculty Research Development fund, and Vodafone New Zealand Ltd. Funders were not involved in the conduct of the study or interpretation of the findings."

X26-i) Comment on ethics committee approval

"Human subjects ethical approval was provided by the Northern Region Y Ministry of Health Human Ethics Committee (NTY/0/09/088). "

x26-ii) Outline informed consent procedures

"Those who wished to participate were given full study information and asked to complete a written consent form and baseline data collection forms immediately after the presentation. "

X26-iii) Safety and security procedures

These are to be fully explained in the main results paper and were not consdiered relevant here

X27-i) State the relation of the study team towards the system being evaluated

"The authors have no conflicts of interest"
